# QSAR-Based Virtual Screening of Natural Products Database for Identification of Potent Antimalarial Hits

**DOI:** 10.3390/biom11030459

**Published:** 2021-03-19

**Authors:** Letícia Tiburcio Ferreira, Joyce V. B. Borba, José Teófilo Moreira-Filho, Aline Rimoldi, Carolina Horta Andrade, Fabio Trindade Maranhão Costa

**Affiliations:** 1 Laboratory of Tropical Diseases Prof. Dr. Luiz Jacintho da Silva, Department of Genetics, Evolution, Microbiology and Immunology, University of Campinas-UNICAMP, Campinas, SP 13083-864, Brazil; ltiburciof@gmail.com (L.T.F.); joycevillaverde@gmail.com (J.V.B.B.); rimoldialine@gmail.com (A.R.); 2 Laboratory of Molecular Modeling and Drug Design, LabMol, Faculty of Pharmacy, Federal University of Goiás, Goiânia, GO 74605-170, Brazil; teofarma1@gmail.com (J.T.M.-F.); andradech@yahoo.com (C.H.A.)

**Keywords:** *Plasmodium falciparum*, natural products, virtual screening, experimental validation, QSAR, ADME

## Abstract

With about 400,000 annual deaths worldwide, malaria remains a public health burden in tropical and subtropical areas, especially in low-income countries. Selection of drug-resistant *Plasmodium* strains has driven the need to explore novel antimalarial compounds with diverse modes of action. In this context, biodiversity has been widely exploited as a resourceful channel of biologically active compounds, as exemplified by antimalarial drugs such as quinine and artemisinin, derived from natural products. Thus, combining a natural product library and quantitative structure–activity relationship (QSAR)-based virtual screening, we have prioritized genuine and derivative natural compounds with potential antimalarial activity prior to in vitro testing. Experimental validation against cultured chloroquine-sensitive and multi-drug-resistant *P. falciparum* strains confirmed the potent and selective activity of two sesquiterpene lactones (LDT-597 and LDT-598) identified *in silico*. Quantitative structure–property relationship (QSPR) models predicted absorption, distribution, metabolism, and excretion (ADME) and physiologically based pharmacokinetic (PBPK) parameters for the most promising compound, showing that it presents good physiologically based pharmacokinetic properties both in rats and humans. Altogether, the in vitro parasite growth inhibition results obtained from in silico screened compounds encourage the use of virtual screening campaigns for identification of promising natural compound-based antimalarial molecules.

## 1. Introduction

Malaria is a mosquito-borne disease transmitted by the bite of Anopheles mosquitoes infected with *Plasmodium* parasites. Mainly caused by *Plasmodium falciparum* and *Plasmodium vivax*, malaria still imposes a heavy burden upon developing countries as it is responsible for high rates of morbidity and mortality. In 2018, the World Health Organization (WHO) recorded 228 million cases of the disease worldwide with an estimate of approximately 400,000 malaria-related deaths [1].

The decreasing rate of cases tracked by the WHO in the last decade has stalled in the last 5 years [1], portraying how fragile the gains achieved can be. The COVID-19 pandemic, which arose in 2019, menaces these gains even further, as the spread of SARS-CoV-2 leads to extra pressure on health systems, especially in low-resourced settings [2]. It is estimated that disruption of malaria control endeavors in Africa due to the COVID-19 pandemic could lead to a malaria burden in 2020 that is the double of that registered in 2019 [3]. Moreover, selection of parasite strains resistant to currently available antimalarial drugs [4] has raised serious concerns about maintaining global achievements in the battle against malaria. The scenario of parasite resistance has threatened the front-line treatment with Artemisinin Combined Therapies (ACTs), as the clinical efficacy of artemisinin (ART) and its derivatives has been compromised by delayed parasite clearance in Southeast Asia [5]. Even more alarming is the recent evidence of *de novo* emergence of *Pf*kelch13-mediated artemisinin resistance in Africa [6]. Together, these reports highlight the compelling call for new therapies based on alternative molecules with potent activity against the malaria parasite, especially drug-resistant *Plasmodium* strains.

The storyline of antimalarial drug development cannot be distinguished from the relevance of biodiversity-derived molecules. One of the first antimalarial indications in history, the use of *Chinchona* decoctions for recurring fevers even before the characterization of malaria parasites [7], motivated the isolation of quinine—an alkaloid with antiplasmodial properties and one of the most representative antimalarial drugs to date. Likewise, the use of *Artemisia annua* decoctions for fever treatment inspired the description of its active principle, artemisinin, which now constitutes the first line of malaria treatment indicated by the WHO [8].

For decades, the discovery of therapeutic molecules derived from natural compounds relied solely on laborious rounds of phenotypic screenings and subsequent cycles of compound purification [9], which is an onerous and uncertain process. However, progress in computational software and the availability of massive online commercial datasets of chemical compounds with their biological properties annotated [10,11] have boosted the development of computer-aided drug design (CADD). In this new setting of drug discovery and development, CADD opens avenues for reducing time and costs associated with identification of new drug candidates and consequent more effective pathways until the workbench [12,13]. Among CADD techniques, quantitative structure–activity relationships (QSARs) have been widely applied to hit identification by virtual screening [14,15,16] in order to predict physicochemical properties and biological activity of molecules. Different QSAR-based virtual screenings have been applied by our group for the effective description of new candidate hits for neglected tropical diseases, including malaria [17,18,19,20]. In light of this, commercial natural products libraries link virtual screening campaigns to the benefits of natural product-based drug discovery. Even though such libraries tend to deliver molecules not synthetically tractable due to their complex scaffolds, their advantages rely on the higher structural and physicochemical diversity, and wider coverage of the chemical space of natural products compared to synthetic drugs [21].

The goal of the present study was to develop a virtual screening pipeline for identifying potent natural products and natural product-derived molecules with antimalarial activity available in a commercial database by integrating QSAR-based virtual screening and further experimental evaluation of chloroquine-sensitive and multi-drug-resistance *P. falciparum* strains, in addition to testing their in vitro therapeutic indexes in human hepatoma cells. Finally, we analyzed multiple absorption, distribution, metabolism, and excretion (ADME) and physiologically based pharmacokinetic (PBPK) parameters to ensure drug efficacy and tolerability.

## 2. Materials and Methods

**Figure 1:**
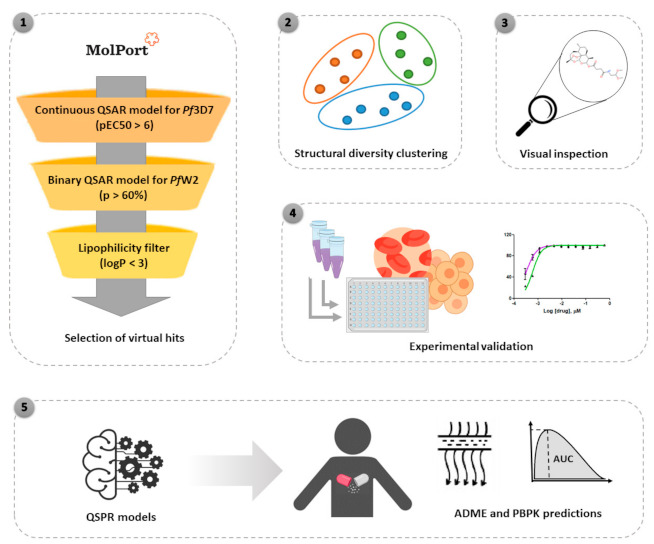
General workflow of the virtual screening of natural compound database followed by experimental validation. The following steps were conducted: (**1**) quantitative structure–activity relationship (QSAR)-based virtual screening of MolPort natural products and derivatives database and selection of compounds with best predicted antimalarial activity; (**2**) analysis of structural diversity by clustering; (**3**) visual inspection; (**4**) experimental validation against *P. falciparum* blood stages and mammalian HepG2 cells and (**5**) quantitative structure–property relationship (QSPR) models for prediction of absorption, distribution, metabolism, and excretion (ADME) and physiologically based pharmacokinetic (PBPK) properties.

The workflow for the study design is summarized in Figure 1.

### 2.1. Virtual Screening and Structural Diversity Clustering

Steps were performed in our in-house QSAR workflow implemented on KNIME 3.2.2 [22]. In-house QSAR models [23] were applied for virtual screening of the natural products branch of MolPort commercial database (www.molport.com, accessed on 3 May 2019). The virtual screening aims to identify, within this database, a subset of compounds with potential biological activity against *P. falciparum* chloroquine-sensitive (3D7) and multi-drug-resistant (W2) strains. Prior to QSAR-based virtual screening predictions, the compounds were curated according to data curation protocols established by Fourches and colleagues [24,25,26]. Then, QSAR models were used to predict the *P. falciparum* 3D7 and W2 inhibition activities of compounds. The in-house QSAR models used for virtual screening were previously developed for 3D7 and W2 strains using learning sets compiled from PubChem database (PubChem IDs for 3D7 dataset: AID_1828, AID_449703, AID_524790, AID_660866 and for W2 dataset: AID_1883, AID_449704, AID_524796, AID_606570). These datasets were compiled and curated according to best practices of QSAR modeling and after curation, the 3D7 learning set contained 7873 compounds (3497 actives and 4376 inactives) and the W2 learning set contained 7403 compounds (3637 actives and 3766 inactives). Both datasets were then used to build consensus models with a Random Forest algorithm and five different molecular descriptors (Avalon, MACCS, Morgan, FeatMorgan and AtomPair) [23]. Three criteria were used for selection of virtual hits: (i) predicted pEC_50_ against *P. falciparum* 3D7 should be ≥6; (ii) probability of activity (p) against *P. falciparum* W2 should be ≥0.6 (p > 60%) and (iii) logP filters were also added to predict good lipophilicity with logP < 3 using XLogP [27]. For prioritization of structurally diverse compounds, molecules predicted to be active by the virtual screening were clustered through the Butina method [28] implemented in Python 3.6 and using the workflow proposed by Sydow and colleagues [29], which groups compounds based on Tanimoto similarity and picks a set of diverse compounds from these groups. Finally, the selected virtual hits were purchased and submitted to in vitro experimental evaluation. The similarity map and was generated using OSIRIS DataWarrior software v.05.02.01 [30].

### 2.2. Compound Preparation

All natural compounds and derivatives selected were purchased from MolPort and dissolved in DMSO at 10 mM. Chloroquine and artesunate (standard antimalarial drugs) were purchased from Sigma-Aldrich (St. Louis, MO, USA) and prepared at 10 mM as well.

### 2.3. Plasmodium falciparum In Vitro Culture

Chloroquine-sensitive (3D7) and multi-drug-resistant (W2) *P. falciparum* strains were cultured following the candle jar method as described by Trager and Jensen [31]. Briefly, parasites were maintained in RPMI 1640 medium supplemented with 10% A+ human plasma at 5% CO_2_ atmosphere. Ring stage synchronized cultures were obtained by two consecutive treatments with a 5% D-sorbitol solution [32] in 48 h intervals.

### 2.4. In Vitro Assays for P. falciparum Growth Inhibition

Parasite growth inhibition assays were conducted by distributing ring stage synchronized parasites at 2% hematocrit and 0.5% parasitemia in 96-well plates (NEST Biotechnology Co., Ltd., Wuxi, Jiangsu, China). Parasites were incubated in the presence of drugs in a two-fold 12-point serial dilution starting at 10 µM in duplicate. Chloroquine was used as an antimalarial standard. At the end of a 72 h incubation, parasitemia was determined by fluorescence reading at 490 nm excitation and 540 nm emission (BMG CLARIOstar, BMG Labtech Inc., Durham, NC, USA) using SybrGreen fluorescent dye as described by Hartwig et al. [33]. Parasite growth inhibition was determined as a percentage relative to drug-free control. EC_50_ values were interpolated from log doses vs. inhibition curves in GraphPad Prism 6 (GraphPad, La Jolla, CA, USA).

### 2.5. Citotoxicity Assays

Cytotoxicity of selected compounds was evaluated using the MTT reduction assay (3-[4,5-dimethyl-thiazol-2-yl]-2,5-diphenyltetrazolium chloride) [34]. Human hepatoma (HepG2) cells were cultured in Dulbecco’s Modified Eagle Medium supplemented with gentamycin (40 mg/L) and 10% heat-inactivated fetal bovine serum at 37 °C and 5% CO_2_. Cells were seeded in 96-well plates (NEST Biotechnology Co., Ltd., Wuxi, Jiangsu, China) at 10^5^ cells per well and incubated in the presence of a serial dilution of the drugs starting at 100 µM. After 72 h of incubation, MTT was added to the wells. The optical density was measured at 570 nm (CLARIOStar, Labtech BMG Inc, Durham, NC, USA) and cell viability was expressed as a percentage relative to the untreated control. CC_50_ values were calculated by plotting a log dose vs. viability curve in GraphPad Prism 6 (GraphPad, La Jolla, CA, USA). The selectivity index (SI) of the compounds was determined by the expression:

SI = (HepG2 CC_50_)/(*Pf* EC_50_),

where HepG2 CC_50_ corresponds to the cytotoxic concentration of compounds in HepG2 cells and *Pf* EC_50_ relates to the 50% inhibitory concentration on *P. falciparum* 3D7 strain.

### 2.6. In Silico Predictions of Metabolism, ADME and PBPK

For the in silico predictions of the compound’s metabolism, we used the web tool BioTransformer [35], which is a software tool that predicts small molecule metabolism in mammals, their gut microbiota, as well as the soil/aquatic microbiota. The program Detoxie^®^ (http://insilicall.com/, accessed on 17 December 2020) was used to predict absorption, distribution, metabolism, and excretion (ADME), and physiologically based pharmacokinetic (PBPK) properties. This software is an artificial intelligence de-risking application based on quantitative structure–property relationship (QSPR) models for each endpoint and human PBPK model.

## 3. Results

### 3.1. QSAR-Based Virtual Screening

**Figure 2:**
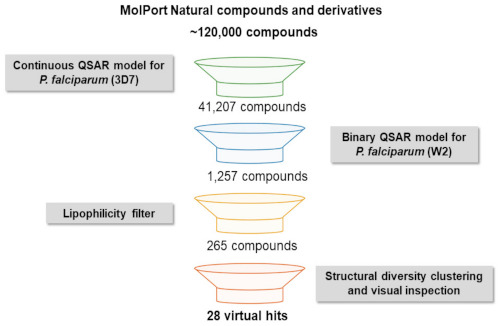
Virtual screening workflow for the identification of natural compounds and derivatives active against *P. falciparum*.

In-house built and validated QSAR models were used for virtual screening, whose results are summarized in Figure 2. The entire MolPort database of commercially available natural products and derivatives containing approximately 120,000 chemical compounds was screened for identification of potentially active compounds against both *P. falciparum* chloroquine-sensitive (3D7) and multi-drug-resistant (W2) strains. Compounds were downloaded and prepared for screening by analyzing their chemical structures according to data curation protocols proposed by Fourches et al. [24,25,26]. Briefly, explicit hydrogens were added, salts were removed and specific chemotypes were normalized. Moreover, polymers, inorganic salts, organometallic compounds and mixtures were also removed. The first filter applied, which predicted activity against *P. falciparum* 3D7 on a continuous QSAR model (pEC_50_ ≥ 6) selected 41,207 compounds. Next, the binary model for activity against multi-drug-resistant *P. falciparum* W2 (p ≥ 0.6) narrowed the list down to 1257 compounds predicted to be active. Once poor physicochemical properties could be a relevant bottleneck in late-stage drug development, we added a filter to select compounds with good lipophilicity (logP < 3), which resulted in 265 compounds being selected after the virtual screening pipeline (Supplemental File S1).

### 3.2. Structural Diversity Clustering

Starting with the 265 virtual hits obtained from screening the MolPort natural products and derivatives database, we performed an analysis of structural diversity based on clustering of structurally similar compounds. The grouping search for a centroid compound which consists of compounds containing bigger amounts of neighbor compounds (or compounds that are structurally similar) and selects 10 of the nearest neighbor compounds to form a cluster. We observed that among more than 120 clusters obtained, more than half contained only one molecule—i.e., the chemical space itself is already diverse (Supplementary Figure S1). Inside each cluster, a structure–activity relationship (SAR) analysis was performed in order to identify the compounds predicted to be the most active within each structural group. Afterwards, just a few compounds were selected from each cluster in order to obtain the most structural diversity possible. Finally, we obtained 188 compounds with significative structural diversity. Among those, 28 were selected by visual inspection.

### 3.3. In Vitro Screening against P. falciparum

**Figure 3:**
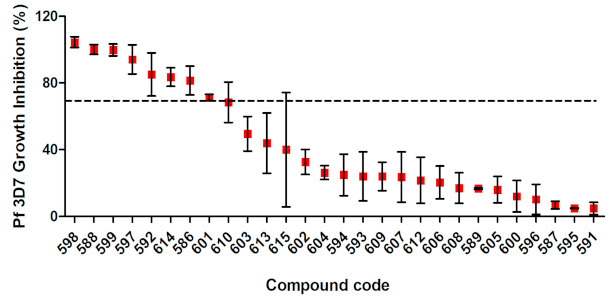
In vitro growth inhibition of asexual blood stage *P. falciparum* (3D7) for prioritized natural compounds and derivatives from virtual screening. The inhibitory potential of different compounds was tested at a concentration of 5 μM and the inhibition of parasitemia was measured after 72 hours of incubation. The dashed line represents the cutoff used to highlight the most promising compounds above 70% of inhibition.

The 28 virtual hits selected were purchased and experimentally evaluated. In order to optimize the screening process, we carried out a preliminary in vitro screen to determine each compound’s ability to inhibit the growth of the chloroquine-sensitive *P. falciparum* 3D7 strain at a concentration of 5 µM (Figure 3). Among the 28 compounds tested, 8 (LDT-598, LDT-588, LDT-599, LDT-597, LDT-592, LDT-614, LDT-586 and LDT-601) inhibited more than 70% of parasite growth at 5 µM. Therefore, we aimed to investigate the 50% inhibitory concentration (EC_50_) of the eight most promising compounds against both chloroquine-sensitive and multi-drug-resistant parasite strains (Table 1).

Table 1 shows the five compounds (LDT-586, LDT-588, LDT-597, LDT-598 and LDT-599) that demonstrated good antimalarial activity with EC_50_ < 5 µM for both *P. falciparum* strains tested, including a multi-drug-resistant one (W2). It is worth noting that compounds LDT-597 and LDT-598 showed remarkable activity with EC_50_ of 0.54 and 0.78 nM, respectively, against *P. falciparum* 3D7 and EC_50_ of 0.56 and 0.59 nM against *P. falciparum* W2.

### 3.4. Cytotoxicity against Human Cells

Cytotoxicity was assessed in human hepatoma cells (HepG2). Among the eight compounds tested, four of them showed favorable therapeutic indexes for hit compounds with an SI > 10 (LDT-586, LDT-588, LDT-597 and LDT-598). Two compounds showed interesting results as they were highly selective: LDT-597 and LDT-598 showed in vitro therapeutic indexes of 33,870.37 and 33,299.1, respectively, likely due to their low nanomolar half maximal inhibitory concentrations (EC_50_) in *P. falciparum* 3D7.

### 3.5. In Silico Predictions of Metabolism, ADME and PBPK

**Figure 4:**
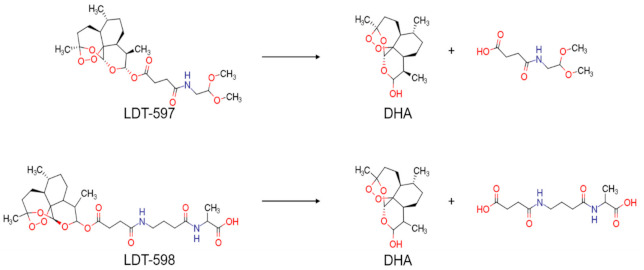
Predicted metabolism of compounds LDT-597 and -598 via plasma carboxylesterases predicted using the software BioTransformer.

We have also analyzed the metabolism of LDT-597 and LDT-598 using the software BioTransformer. The program showed that the carboxylic esters of these compounds might be hydrolyzed into dihydroartemisinin (DHA) (Figure 4) by plasma carboxylesterases, showing the same type of metabolization of artesunate [36].

**Figure 5:**
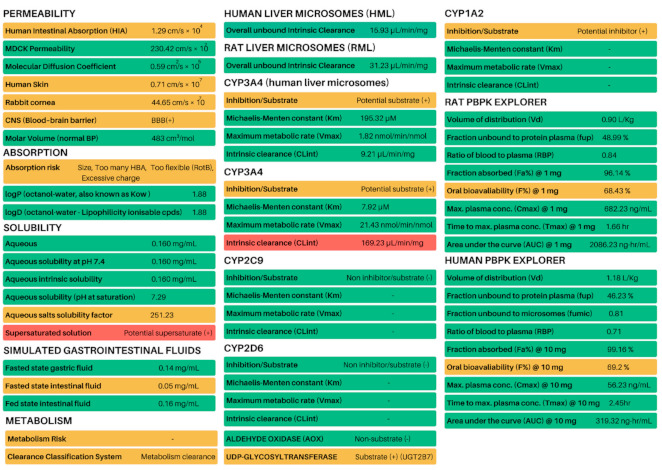
ADME and PBPK multiparametric prediction of LDT-597 using the Detoxie^®^ software (http://insilicall.com/, accessed on 17 December 2020).

Artesunate has significantly greater solubility in water than either artemisinin, dihydroartemisinin or artemether, which influences its diffusion across mucosal membranes as well as other pharmacokinetic properties [37]. For this reason, we decided to compare the in silico ADME and PBPK profiles of LDT-597 (Figure 5), Artesunate and DHA (Supplemental Figures S2 and S3, respectively) using the program Detoxie® which is an artificial intelligence de-risking application based on QSPR models for each endpoint and human PBPK model. While compound LDT-597 was predicted to be slightly more soluble than artesunate and a lot more soluble than DHA, its predicted intestinal permeability is 1.29 cm/s × 10^4^, while artesunate’s predicted intestinal permeability is 2.29 cm/s × 10^4^. Moreover, the volume of distribution (VD) of compound LDT-597 was considerably higher than that of artesunate. As compound LDT-597 showed to have good physiologically based pharmacokinetic properties both in rats and humans, it was predicted to have a higher fraction unbound to protein plasma (UF) when compared to artesunate and DHA, which results in better compound bioavailability.

## 4. Discussion

Considering the relevance of natural products and derivatives for antimalarial drug development, we have applied a QSAR-based virtual screening pipeline for identification of potent antimalarial candidates against chloroquine-sensitive (3D7) and multi-drug-resistant (W2) *P. falciparum* strains. Firstly, the MolPort database for natural compounds and derivatives was screened with the aim of identifying molecules with predicted EC_50_ against *P. falciparum* 3D7 ≤ 1 µM and predicted probability of being active against *P. falciparum* W2 > 60%. These molecules were also passed by a lipophilicity prediction filter and were verified for agreement within the applicability domain of QSAR models used in this study. It is important to note that we did not use a molecular weight filter due to large carbonic chains in natural products and derivatives, which could be a huge restriction and lead to loss of potential candidate molecules. The selected virtual hits were further divided into structurally diverse clusters and visually inspected for compound prioritization. Experimental in vitro assays against intraerythrocytic *P. falciparum* 3D7 and W2 strains were conducted to pinpoint active compounds.

Among the compounds evaluated, some of them do not show impressive phenotypic activity (high EC_50_ values and consequent low in vitro therapeutic indexes), which might be due to the limited size of natural products-based commercial databases compared to those of synthetic drugs. Even though the universe of natural products covers a more diverse chemical space, it is limited by being heavily dependent on the identification, isolation, and characterization of bioactive natural products from the biodiversity. However, our QSAR models were able to identify compounds with relevant activity in vitro, as highlighted by the nanomolar levels of parasite growth inhibition for compounds LDT-597 and LDT-598 against multi-drug-resistant *P. falciparum* W2 strains. These two compounds, which are artesunate derivatives, show activity about 10-fold more potent than artesunate itself (EC_50_ = 5.7 nM) against this same multi-resistant strain [38]. The identification of sesquiterpene lactones containing an endoperoxide bridge as the most promising antimalarial candidates within a diverse set of natural products and derivatives illustrates the agreement in the results obtained with the virtual screening pipeline, as this class of compounds has been widely characterized as potent antimalarial molecules [39,40,41]. By describing structural moieties that support antimalarial activity, HQSAR analysis performed by Avery and colleagues [42] showed that the lactone ring has a strong positive contribution for the antimalarial activity of artemisinin. Once this lactone ring is the main common substructure among artemisinin, artesunate and compounds LDT-597 and LDT-598, the robust potency of the latter two compounds can be explained by this shared feature that is responsible for antimalarial activity even against drug-resistant *P. falciparum* parasites.

Since the pace of drug resistance selection in *P. falciparum* parasites has been as fast as ever, there is an urgent need to ensure efficacy of compounds that stand out within the pipeline of drug discovery. For this reason, in silico models for pharmacokinetic antimalarial drugs have been developed, including those that have analyzed properties of artemisinin derivatives [43,44,45]. Altogether, the results highlight a better pharmacokinetics profile of LDT-597 when compared with artesunate, which might enhance efficacy of treatments using this compound instead of the traditional artemisinin derivatives.

## 5. Conclusions

Herein, we report a QSAR-based virtual screening study for predicting the antimalarial potential of a library of natural compounds and derivatives and explore the potential molecular target for the most potent compounds. Aiming to validate our in silico approach, we identified two compounds with low nanomolar inhibition levels against multi-drug-resistant *P. falciparum* strain in vitro. Moreover, in silico ADME/PBPK analysis of one of these compounds showed that it presents favorable physicochemical characteristics. The work presented endorses the applicability of natural compound databases for virtual screening campaigns and supports QSAR as a means of identifying potent and selective compounds against the malaria parasite.

## Figures and Tables

**Table 1:** The most promising compounds predicted to be active against *P. falciparum* asexual stages selected by virtual screening.

Compound Code (MolPort ID)	2D Structure (Biological Source of NP Precursor)	EC_50_^a^ (µM)		CC_50_^b^ (µM)	In vitro Therapeutic Index ^c^
		*Pf*3D7	*Pf*W2	HepG2	
**LDT-586** **(MolPort-001-745-423)**	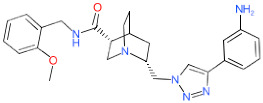 (No data available)	4.52 ± 0.91	3.44 ± 1.30	98.59 ± 0	21.81
**LDT-588** **(MolPort-000-651-065)**	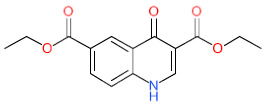 (No data available)	3.84 ± 1.45	2.09 ± 1.17	67.04 ± 2.28	17.46
**LDT-592** **(MolPort-002-323-504)**	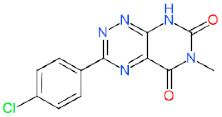 (No data available)	9.09 ± 3.74	3.11 ± 0.54	17 ± 0	1.87
**LDT-597** **(MolPort-001-732-360)**	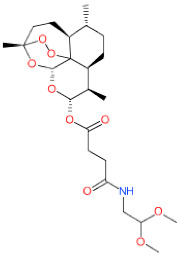 (*Artemesia annua*)	0.0005 ± 0.00	0.0005 ± 0.00	18.29 ± 3.51	33,870.37
**LDT-598** **(MolPort-001-732-370)**	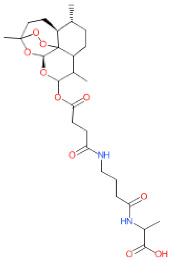 (*Artemesia annua*)	0.0007 ± 0.00	0.0006 ± 0.00	25.94 ± 1.13	33,299.10
**LDT-599** **(MolPort-001-737-485)**	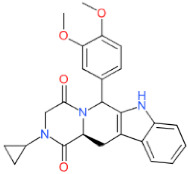 (*Aspergillus fumigatus*)	3.68 ± 1.92	2.74 ± 0.78	20.96 ± 2.51	5.70
**LDT-601** **(MolPort-002-506-405)**	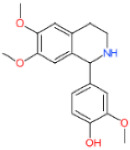 (*Pachycereus weberi*, *Pachycereus pringlei*, *Pachycereus pecten -aboriginum*, *Backebergia militaris* and *Carnegiea gigantea*)	6.61 ± 3.20	0.65 ± 0.47	21.79 ± 4.16	3.30
**LDT-614** **(MolPort-044-180-513)**	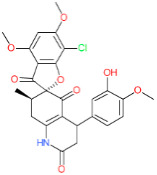 (*Penicillium patulum*)	5.26 ± 0.52	5.65 ± 3.11	23.55 ± 1.82	4.48
**Chloroquine**		0.0079 ± 0.00	0.147 ± 0.04	ND	-
**Artesunate**		0.0016 ± 0.00	ND	ND	-

^a^ EC_50_: half of the maximum inhibitory concentration in 3D7 and W2 strains and their respective standard deviations; ^b^ CC_50_: half the maximum cytotoxic concentration in HepG2 cells; ^c^ SI: Selectivity index calculated from CC_50_*/*EC_50_ (3D7). NP: natural products. ND: not determined. The data derive from at least two independent experiments.

## Data Availability

Data is contained within the article or supplementary material. The data presented in this study are available in supplementary material.

## Supplementary Materials

The following are available online at https://www.mdpi.com/2218-273X/11/3/459/s1, Supplemental File S1: Virtual screening of selected hits. Supplemental Figure S1: Structural diversity map of 265 virtual hits obtained from the virtual screening. Compounds were clustered based on their Tanimoto similarity. Grey circles show compounds that were removed during the clusterization process; these compounds mostly belong to bigger clusters where the structural diversity is small. The green circles and rectangles show compounds that were kept by the clusterization algorithm. Green rectangles are the compounds selected from different clusters for visual inspection and acquisition. Supplemental Figure S2: ADME and PBPK multiparametric prediction of artesunate using the Detoxie^®^ software. Supplemental Figure S3: ADME and PBPK multiparametric prediction of dihydroartemisinin using the Detoxie^®^ software.
