# Supplementary figures and images for: QSAR-Based Virtual Screening of Natural Products Database for Identification of Potent Antimalarial Hits
Source: Biomolecules. 2021 Mar 19;11(3):459. doi: 10.3390/biom11030459 (PMC8003391; doi:10.3390/biom11030459)

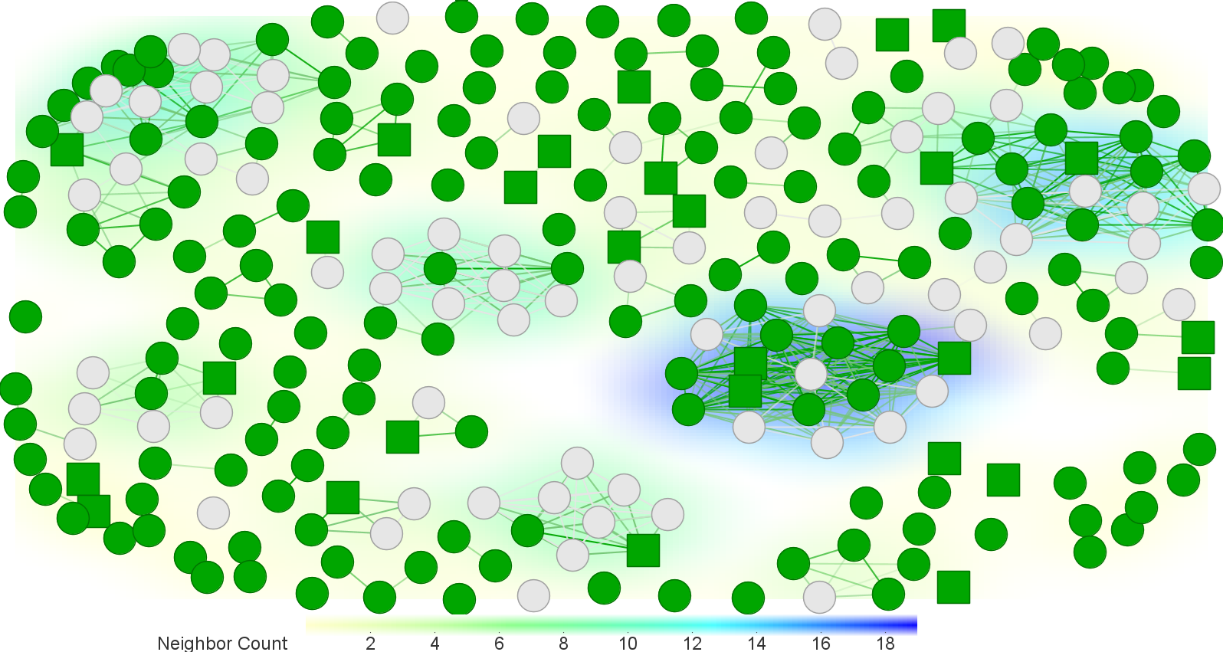

Supplement: Supplementary file 1 [file biomolecules-11-00459-s001.zip › Supplemental Figure 1 - Structural diversity map of 265 virtual hits obtained from the virtual screening.png]

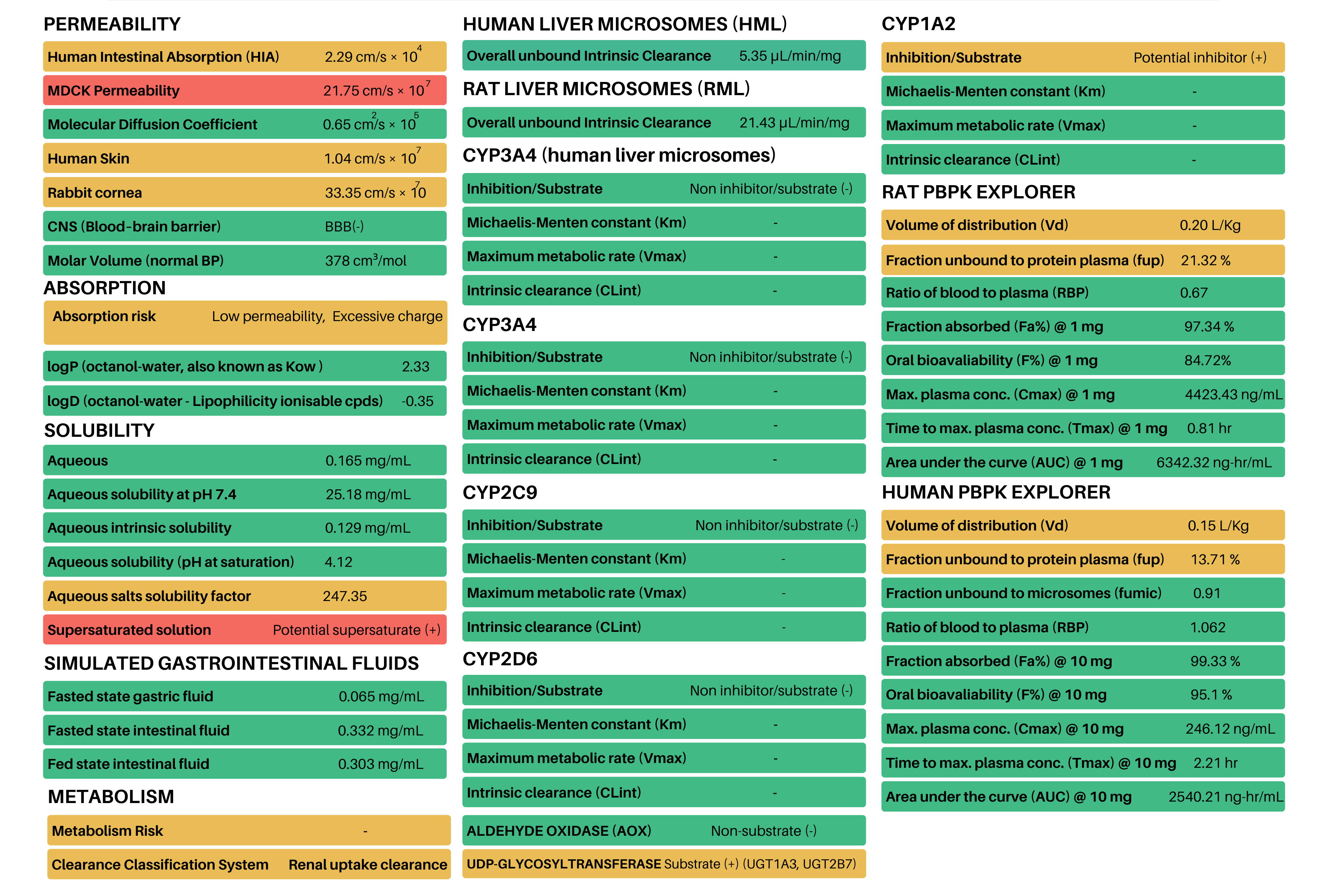

Supplement: Supplementary file 1 [file biomolecules-11-00459-s001.zip › Supplemental Figure 2 - ADME and PBPK multiparametric prediction of artesunate using the Detoxie® software.png]

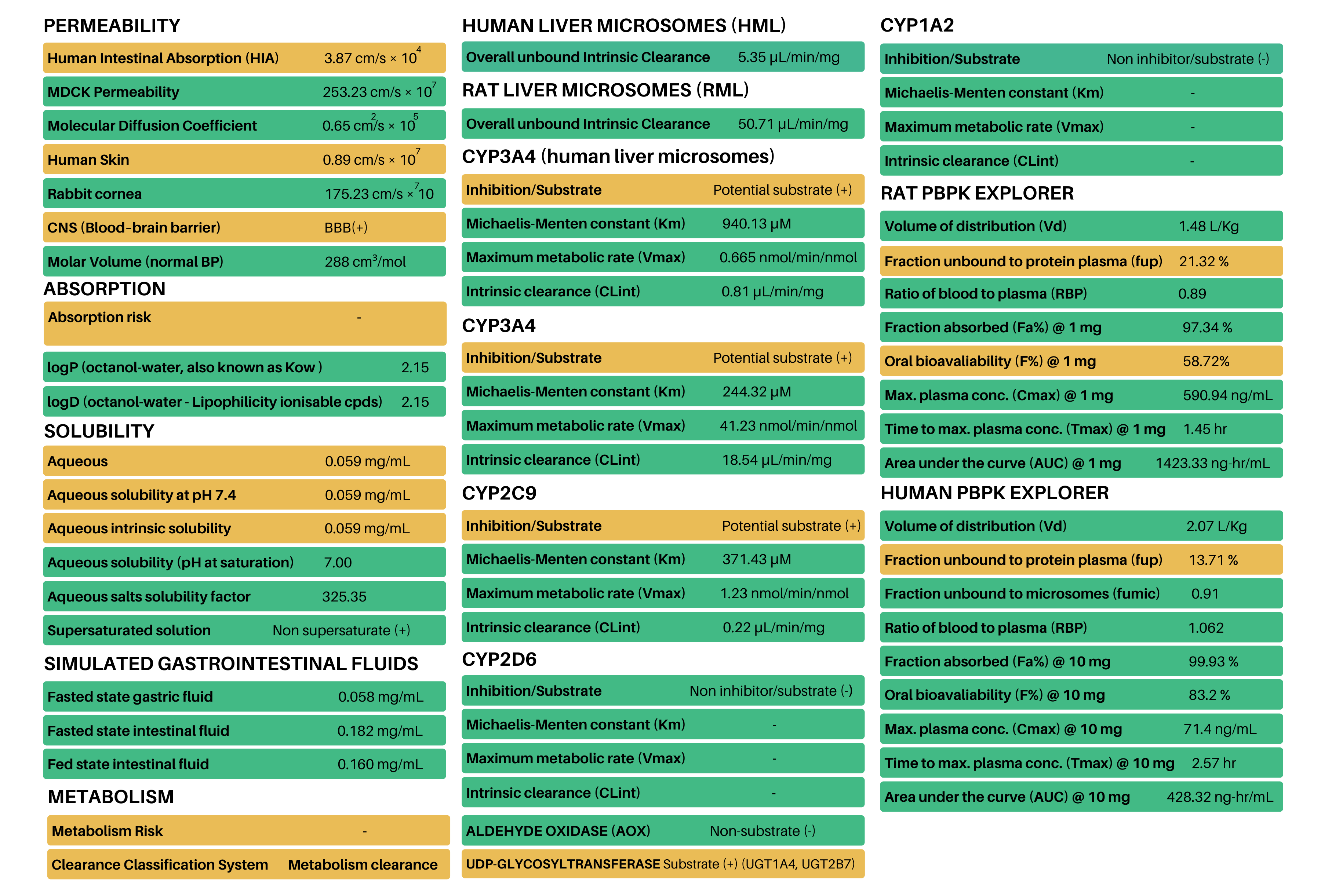

Supplement: Supplementary file 1 [file biomolecules-11-00459-s001.zip › Supplemental Figure 3 - ADME and PBPK multiparametric prediction of dihydroartemisinin using the Detoxie® software.png]
